# Precise antibacterial therapeutics based on stimuli-responsive nanomaterials

**DOI:** 10.3389/fbioe.2023.1289323

**Published:** 2023-10-18

**Authors:** Ziqi Wu, Ran Nie, Yao Wang, Qihui Wang, Xiang Li, Yuguang Liu

**Affiliations:** Department of Stomatology, The First Hospital of Jilin University, Changchun, China

**Keywords:** stimuli-responsive, nanomaterials, antibacterial, exogenous stimuli, microenvironment stimuli

## Abstract

Bacterial infection refers to the process in which bacteria invade, grow, reproduce, and interact with the body, ultimately causing a series of pathological changes. Nowadays, bacterial infection remains a significant public health issue, posing a huge threat to human health and a serious financial burden. In the post-antibiotic era, traditional antibiotics are prone to inducing bacterial resistance and difficulty in removing bacterial biofilm. In recent years, antibacterial therapy based on nanomaterials has developed rapidly. Compared with traditional antibiotics, nanomaterials effectively remove bacterial biofilms and rarely result in bacterial resistance. However, due to nanomaterials’ strong permeability and effectiveness, they will easily cause cytotoxicity when they are not controlled. In addition, the antibacterial effect of non-responsive nanomaterials cannot be perfectly exerted since the drug release property or other antibacterial effects of these nano-materials are not be positively correlated with the intensity of bacterial infection. Stimuli-responsive antibacterial nanomaterials are a more advanced and intelligent class of nano drugs, which are controlled by exogenous stimuli and microenvironmental stimuli to change the dosage and intensity of treatment. The excellent spatiotemporal controllability enables stimuli-responsive nanomaterials to treat bacterial infections precisely. In this review, we first elaborate on the design principles of various stimuli-responsive antibacterial nanomaterials. Then, we analyze and summarizes the antibacterial properties, advantages and shortcomings of different applied anti-bacterial strategies based on stimuli-responsive nanomaterials. Finally, we propose the challenges of employing stimuli-responsive nanomaterials and corresponding potential solutions.

## Graphical Abstract

**Figure:**
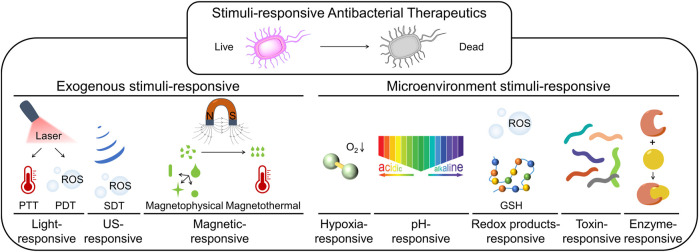
Stimuli-responsive nanomaterials for precise antibacterial therapeutics.

## 1 Introduction

Bacterial infection refers to the process of bacteria invading the host body and causing pathological changes (Taylor et al., 2002; Cossart and Sansonetti, 2004). Nowadays, bacterial infection remains a significant security threat to human society and brings a heavy financial burden (Flores-Mireles et al., 2015; Kamaruzzaman et al., 2017). Bacterial infection leads to the occurrence and development of a variety of diseases and bring pain and inconvenience to patients (White et al., 2003; Wang et al., 2004; Marima et al., 2018; Brouwer et al., 2020). For example, there are a large number of bacteria in the oral cavity, and some bacteria will transform into pathogenic bacteria when the host immune response is weak, which causing damage to the teeth, mucous membrane or bone tissue in the oral cavity, and finally seriously affecting the normal life of patients (Jiang et al., 2021). Antibiotic treatment is essential antibacterial therapy. However, antibiotics only target a small number of bacterial targets, making it easy for bacteria to resist antibiotics (Lambert, 2005; Andersson and Hughes, 2010). The emergence of drug-resistant bacteria has increased the difficulty of curing bacterial infection diseases, forcing humans to find new ways to combat bacterial infection. Besides, bacteria usually form bacterial biofilms to counteract immune clearance and antibiotics within the host (Davies, 2003). Therefore, in the post-antibiotic era, scientists need to develop new antibacterial agents that are efficient in killing bacteria, less susceptible to bacterial resistance, and can clear biofilms.

With the rapid development of modern science and technology, nanomaterials have gradually become one of the research hotspots. Nanomaterial means that the size of the material is between 1 and 100 nm, and its surface and volume properties are significantly different from that of ordinary materials (Kreyling et al., 2010). Nanomaterials have the characteristics of small size, large specific surface area, high reactivity and unique optical, electrical and magnetic properties (Khan et al., 2019). Nanomaterials have been widely developed for disease diagnosis, treatment, and prognosis (Yu et al., 2023). Compared with antibiotics, nanomaterials have multiple passive and extensive antibacterial effects, which makes it difficult for bacteria to develop tolerance to nanomaterials (Hu et al., 2021). Nanomaterials also utilize their high permeability to penetrate bacterial biofilms and clear biofilm substrates through various exogenous and chemical properties (Cui et al., 2022). Many antibacterial strategies based on nanomaterials have been applied in the treatment of bacterial infections, such as photothermal therapy (PTT), photodynamic therapy (PDT), sonodynamic therapy (SDT), chemodynamic therapy (CDT), and gas therapy (Quinn et al., 2015; Chen et al., 2020; Roy et al., 2021; Jia et al., 2022; Thomas-Moore et al., 2022).

Despite the powerful antibacterial effects of nanomaterials, non-responsive nanomaterials cause toxicity to tissue cells and insufficient antibacterial properties in complex infectious microenvironments. Specifically, for direct antibacterial nanomaterials, the antibacterial effects of them are generally non-selective, and if these nanomaterials are not controlled for combating bacteria, they will inevitably damage normal tissues and cells. For nano-delivery systems, uncontrolled drug delivery leads to drug release at inappropriate times and locations, which results in toxicity to normal tissue cells and insufficient drug in lesion sites (Huang et al., 2022a). Therefore, it is necessary to install controllable switches for nanomaterials to achieve precise antibacterial therapeutics. Stimuli-responsive antibacterial nanomaterials can be selectively activated by exogenous or microenvironment stimuli (Canaparo et al., 2019). Stimuli-responsive nanomaterials for antibacterial directly would be activated by exogenous or microenvironment stimuli, generating reactive oxygen species (ROS) and heat or releasing antibacterial agents to eliminate bacteria (Zhang et al., 2023). Once nanomaterials achieve desired antibacterial effects, the operators immediately switch off the stimuli sources to prevent the nanomaterial from continuing to exert functions to damage normal tissues and cells. Stimuli-responsive nano-delivery systems intelligent release antibacterial agents according to disease progression and changes in infectious microenvironment.

Hence, this review aims to enhance the understanding of stimuli-responsive antibacterial nanomaterials. First, we briefly introduce antibacterial nanomaterials based on stimuli-responsive strategies. Then, we summarize the design principles of nanomaterials on the basis of different stimuli-responsive mechanisms. Afterwards, we provide a detailed introduction to the applications of stimuli-responsive antibacterial nanomaterials. Finally, we propose perspectives and suggestions on the future development of antibacterial nanomaterials based on stimuli-responsive strategies. We hope scientists better understand and develop stimuli-responsive nanomaterials for sterilization via this review.

## 2 Design of stimuli-responsive nanomaterials

Exogenous stimuli-responsive nanomaterials mainly include light-responsive, US-responsive, and magnetic-responsive nanomaterials (Karimi et al., 2016). The working mechanism of these nanomaterials is to convert the energy of external stimulation into ROS, heat and other changes of form or state to combat bacteria (Lee et al., 2020; Li et al., 2021a; Um et al., 2021). Although stimuli-responsive antibacterial therapy is based on the unique properties of materials, the design of exogenous stimuli-responsive nanomaterials can be further optimized and improved. For example, changes in the size, shape, structure, and aggregation of nanomaterials significantly affect their functions. Thus, nanomaterials need to be designed and synthesized into types that can maximize their effects (Hotze et al., 2010). Besides, the different functions of nanomaterials require synergy. For instance, the solid bactericidal effect of ROS and the clearing biofilm matrix effect of PTT can be combined for synergistic antibacterial treatment (Hu et al., 2022a). In addition, the generation of ROS in PDT and SDT processes relies on sufficient oxygen, but oxygen in the infected microenvironment is not enough (Tan et al., 2021; Dong et al., 2023a). Therefore, nanomaterials based on PDT and SDT need to be designed to generate oxygen in the infected microenvironment or carry oxygen themselves or generate other free radicals that are not dependent on oxygen. Moreover, the antibacterial effects of many nanomaterials are double-edged swords, and these non-targeted effects may cause damage to normal tissues and cells. Therefore, nanomaterials need to be designed with targeted or on-demand drug delivery capabilities to reduce their toxic effects on tissues. Meanwhile, researchers need to explore the safety thresholds that the body can withstand towards various effects from nanomaterials, which is conducive to optimizing the design of nanomaterials from a biomedical perspective.

Microenvironment-responsive nanomaterials are mainly based on the interaction between nanomaterials and stimuli in the infectious microenvironment, such as hypoxia, low pH, redox products, toxins, and enzymes (Hu et al., 2022b). The design of microenvironment-responsive nanomaterials requires the introduction of nanomaterials that are altered in exogenous and chemical characteristics (like structural modification, hydrophobicity, size, surface charge, and chemical bond) by these stimuli. Scientists further utilize these physicochemical changes to improve nanomaterials' targeting, antibacterial, and drug-release abilities. For example, targeting antibacterial nanomaterials can be designed by introducing charge reversible stimuli-response materials, and nano drug loading systems with controlled release ability can be designed by introducing hydrophobicity changeable or chemical bond breakable stimuli-response materials (Chen et al., 2017; Zhang et al., 2020). The design of microenvironment-responsive nanomaterials should fully consider the interaction between various stimuli and materials in the microenvironment; meanwhile, scientists should consider interferences in the microenvironment because the infection microenvironment is very complex.

In a word, these stimuli-responsive nanomaterials should be designed to remain turn off without external exogenous stimuli or microenvironmental stimuli while immediately keeping turn on with external exogenous stimuli or microenvironmental stimuli. Specifically, different states of exogenous stimuli-responsive nanomaterials should be controlled by operators via regulating the sources of light, ultrasound (US), and magnetic field. When the number of bacteria decreases by more than two logarithms or exogenous stimuli exceeds the tolerance of the body, the operator should stop stimulation. Various states of microenvironmental stimuli-responsive nanomaterials should be spontaneously regulated by the concentration of stimuli, which reflect the degree of bacterial infection. The schematic diagram of stimuli-responsive antibacterial nanomaterials is summarized in Graphical abstract.

## 3 Applications of precise antibacterial therapeutics based on stimuli-responsive nanomaterials

### 3.1 Exogenous stimuli-responsive strategy

Numerous exogenous stimuli can activate nanomaterials to eliminate bacteria, such as light, US, and magnetic (Allafchian and Hosseini, 2019; Yu et al., 2022; Bag et al., 2023). Under the stimulation of these external stimuli, stimuli-responsive nanomaterials mainly generate heat or ROS, which have broad-spectrum and efficient bactericidal ability (Cheng et al., 2023a). The merit of this strategy is that the operator can adjust the intensity and duration of treatment by controlling the switch of the machine that generates the stimulus.

#### 3.1.1 Light-responsive strategy

##### 3.1.1.1 PDT

**FIGURE 1:**
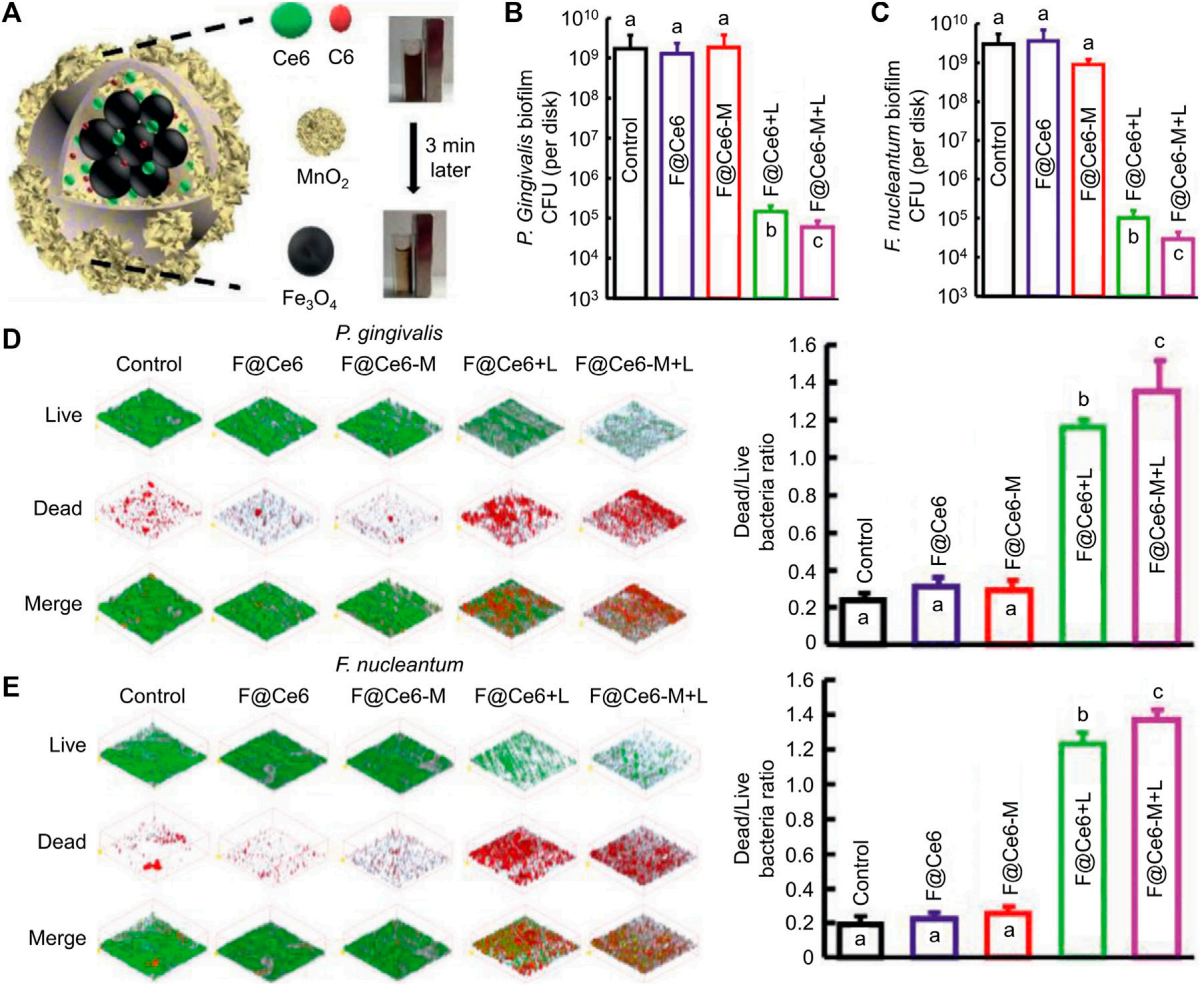
**(A)** schematic diagram of the microstructure and composition of F@Ce6-M. **(B–C)** Corresponding statistical data of colonies of different biofilms. **(D–E)** Live/Dead images and corresponding statistical data of colonies of different biofilms (Sun et al., 2021; Sun et al., 2021). Copyright 2021 Wiley-VCH.

Photosensitizers (PSs), light, and oxygen are the three most crucial elements of PDT (Rodrigues and Correia, 2022). PSs are divided into organic and inorganic PSs, organic PSs like indocyanine green (ICG) have the advantage of high efficiency in producing ROS, while inorganic PSs like TiO_2_ have the advantages of good biosafety and stability (Yuan et al., 2020; Liu et al., 2022; Cheng et al., 2023b). Lights separate into ultraviolet, visible light, and near-infrared light according to the increased wavelength of light (Ji et al., 2017). The penetration depth of light deepens with the increase of wavelength; moreover, ultraviolet light harms organisms and even induces skin cancer. Therefore, near-infrared light is more conducive to treating bacterial infection (Qi et al., 2019; Fang et al., 2022). The oxygen content is closely related to ROS production (Teng et al., 2023). However, there is often a state of hypoxia in bacterial infection microenvironments. Thus, how to increase ROS production under low oxygen conditions has attracted scientists' attention (Bjarnsholt et al., 2022). Sun *et al.* developed an advanced nanocomposite (named F@Ce6-M) that considered the above three crucial elements, which consisting of Fe_3_O_4_, MnO_2_, Chlorin e6 (Ce6), and Coumarin 6 (Figure 1A) (Sun et al., 2021). Ce6 is an efficient organic PSs, but it cannot be stimulated by near-infrared light. Because of the red-shifted absorption induced by increased conjugate structure, the nanoplatform could achieve the PDT effect under near-infrared light, and it is beneficial for killing the biofilm deep in the periodontal pocket. MnO_2_ could generate oxygen via a high level of H_2_O_2_ in the periodontitis environment. The number of bacteria decreased by four logarithms after the therapy of nanoparticles (NPs) (Figures 1B, C), and the biofilm was significantly damaged (Figures 1D, E). Thanks to its excellent antibacterial effect, the number of inflammatory cells and the levels of inflammatory factors in periodontal tissue were significantly reduced. After co-culture of nanocomposites and L929 cells for 24 h, when the concentration of Ce6 in F@Ce6-M was 0–5 μM, the relative survival rate of L929 cells was more than 90%, which suggested that F@Ce6-M had good cytocompatibility. Although PDT has many advantages, it also has shortcomings to be addressed, such as short action radius of ROS (Gao et al., 2021).

##### 3.1.1.2 PTT

**FIGURE 2:**
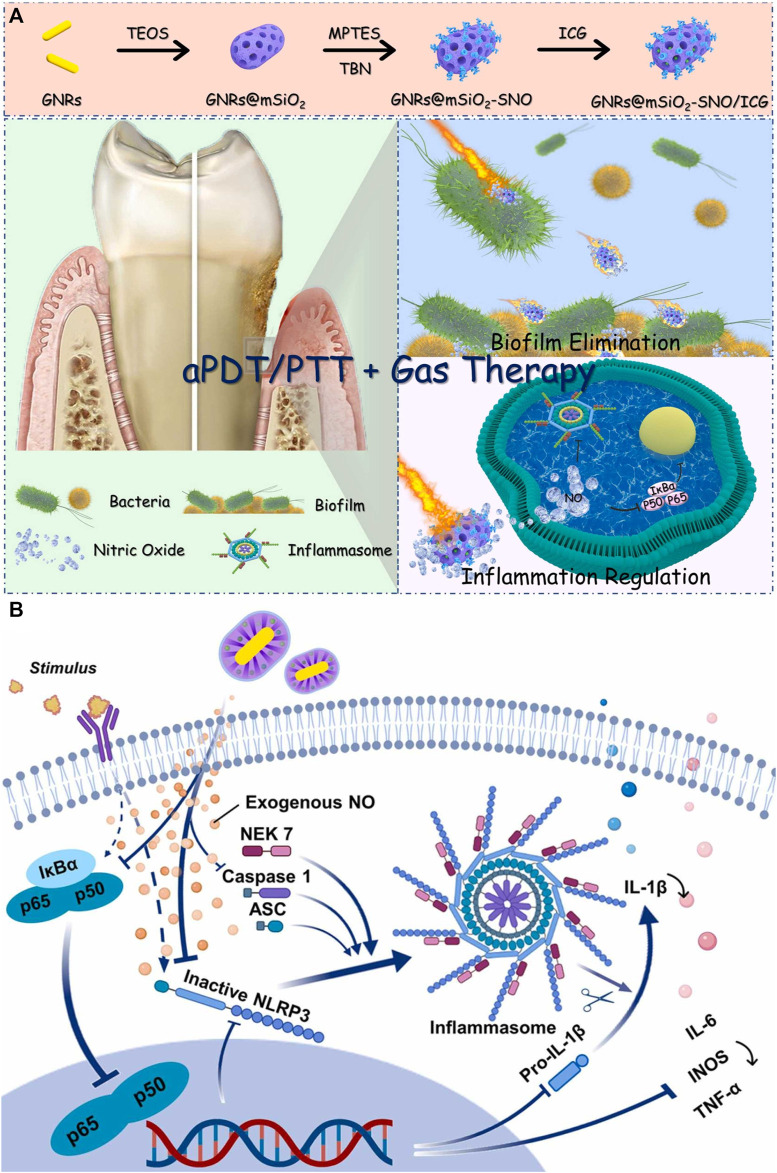
**(A)** Schematic illustration of GNRs@mSiO_2_-SNO/ICG and its anti-biofilm and anti-inflammatory functions for periodontitis treatment. **(B)** Schematic diagram of GNRs@mSiO_2_-SNO/ICG for regulating inflammation (Qi et al., 2022). Copyright 2022 American Chemical Society.

Photothermal agents transfer light energy into thermal energy to exert the PTT process (Jung et al., 2018). Compared to PDT with higher sterilization efficiency towards planktonic bacteria, PTT has higher anti-biofilm efficiency. Since the biofilm is rich in extracellular DNA, polysaccharides, proteins, and other biofilm matrices, which can be cleared by a large amount of heat from PTT, when the biofilm matrix is not protected, the planktonic bacteria are more likely to be killed (Buzzo et al., 2021; Qi et al., 2022). Besides, PTT has been proven to promote vascular regeneration, which facilitates infected wound healing (Wang et al., 2021a; Huang et al., 2022b; He et al., 2023). Among inorganic materials, Au nanorod has a high photothermal conversion rate, adjustable absorption wavelength, and low cytotoxicity (He et al., 2022). Among organic materials, polydopamine not only has a high photothermal conversion rate but also has the merits of being easy to synthesize and connect with other functional groups (Hu et al., 2016). To solve the problem of low sterilization rate of pure PTT, researchers always combine PTT and other strategies like PDT and gas therapy. Qi and her co-workers designed a nanocomposite composed of Au rods, PSs (ICG), and nitric oxide donor (S-nitrosothiols) (Qi et al., 2022), PTT effect destroyed biofilm and promoted S-nitrosothiols to generate nitric oxide (Figure 2A). Nitric oxide could disperse biofilm and relieve inflammation via inhibiting NF-kB signaling pathways (Figure 2B). Toxicity tests *in vivo* by H&E analysis showed the nanocomposites would not result in histological abnormalities or local inflammation of the main organs even after 42 d post-treatment, which proved that the nanocomposites had good histocompatibility. Cheng *et al.* proved that polydopamine could not only achieve the PTT effect but also transfer light energy into TiO_2_ to realize the PDT effect (Cheng et al., 2023b). This simple and stable nanoplatform had rich antibacterial functions, and it reduced the number of bacteria in the biofilm by 99%. The wound caused by bacterial infection almost completely healed 10 days after treatment. Although PTT has been widely used in antibacterial therapy, excessive temperature cause damage to tissues and cells. Studies have shown that the temperature of PTT should be controlled below 45 °C (Dong S. et al., 2023).

#### 3.1.2 US-responsive strategy

**FIGURE 3:**
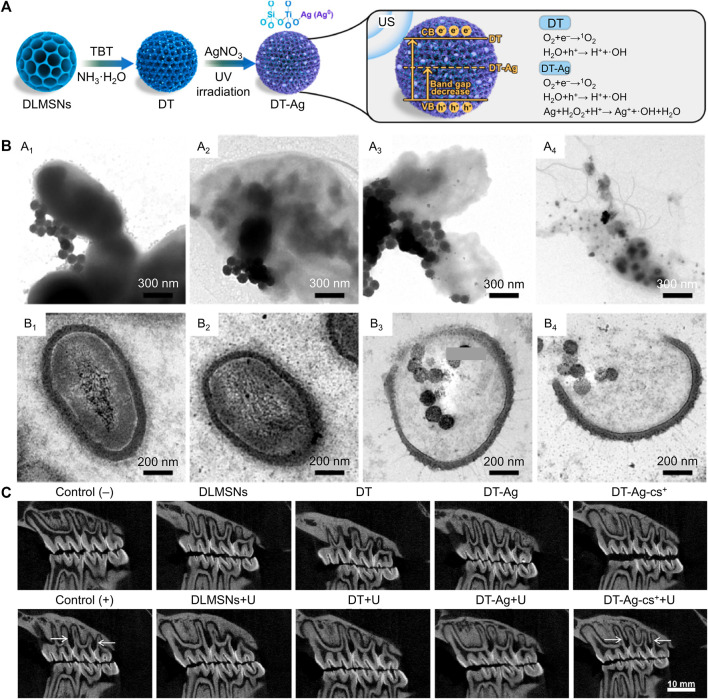
**(A)** Schematic illustration for the synthetic path and SDT mechanism of the nanocomposite. **(B)** TEM images of bacteria after treated by the nanocomposite. **(C)** *In vivo* anti-periodontitis properties of this nanocomposite (Xin et al., 2023; Xin et al., 2023). Copyright 2023 Elsevier.

US can activate sonosensitizers to produce ROS via sonochemistry, sonoporation, pyrolysis, and sonoluminescence (He et al., 2019). The penetration distance of US into tissue is more profound than that of near-infrared light, and the US is a non-invasive stimulus. Hence, SDT based on US has received widespread attention recently (Wu et al., 2022). Currently, relatively few materials have been proven to achieve the SDT effect. TiO_2_ is a fundamental sonosensitizer, but the ROS generation efficiency of TiO_2_ is insufficient due to its wide bandgap (Ning et al., 2022). To settle this critical problem, researchers have attempted to modify TiO_2_ with other materials. For example, Yang’s group added Ag into TiO_2_ to obtain a robust ROS production nanocomposite (Xin et al., 2023). The bandgap of this nanocomposite was obviously reduced after Ag modification, and the ROS yield of this nanocomposite was approximately three times that of pure TiO_2_ (Figure 3A). Besides, the authors also utilized positively charged chitosan to coat this nanocomposite for targeting bacterial cell walls via the electrostatic attraction effect. This nanocomposite with targeting ability could enter the inside of bacteria and synergistic ROS to eliminate bacteria (Figure 3B). *In vivo* experiments showed significant inhibition of alveolar bone loss caused by bacterial infection (Figure 3C). Moreover, the nanocomposite showed slightly cytotoxicity toward different cells after co-cultured for 3 and 5 d at the high concentrations of 400 μg/mL. SDT is an emerging antibacterial therapy, but currently, there are very few sonosensitizers available for SDT, and the mechanism of SDT production is also controversial. Future studies had better focus on developing more advanced sonosensitizers or exploring the mechanism of SDT in detail.

#### 3.1.3 Magnetic-responsive strategy

##### 3.1.3.1 Magnetophysical therapy

**FIGURE 4:**
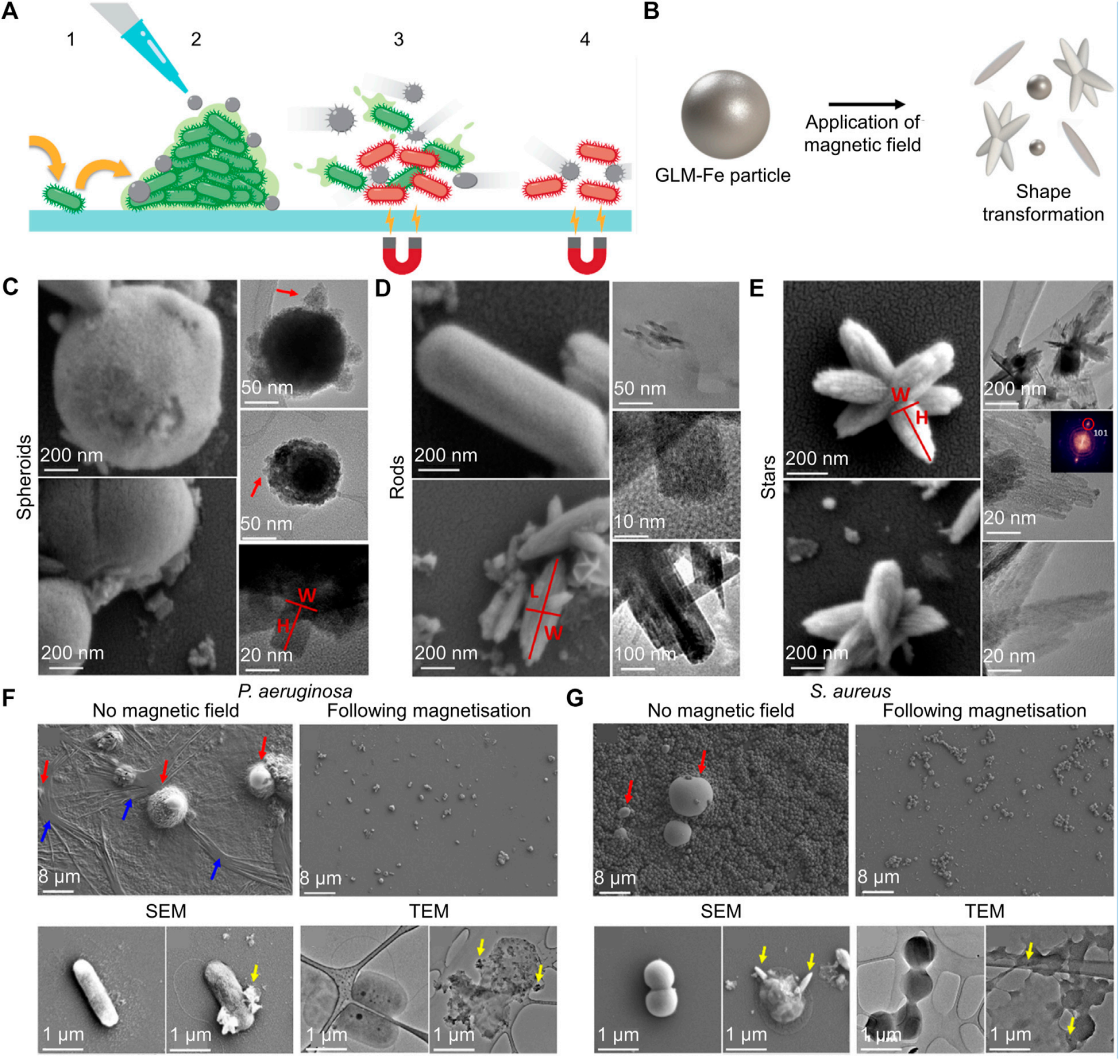
**(A)** Schematic illustration of the antibiofilm properties of the Ga-Fe metal droplets particles. **(B)** Schematic diagram of Ga-Fe metal droplets particles undergoing deformation in a magnetic field. **(C–E)** Ga-Fe droplets transform into spheroids, nanorods, and nanostars in a magnetic field. **(F–G)** SEM and TEM images of various biofilm and bacteria cells after the therapy of Ga-Fe droplets (Elbourne et al., 2020). Copyright 2020 American Chemical Society.

Under a magnetic field, some metal materials will undergo changes in morphology or motion, which can be used to break the structure of bacterial cell membranes and biofilms. For example, Zhou’s group designed glucose oxidase (GOx)-modified Fe_3_O_4_ magnetic NPs (named GMNPs) to ameliorate bacterial biofilm-induced persistent endodontic infections (Ji et al., 2021). The bacterial biofilm matrix was obviously destroyed via the movement of Fe_3_O_4_ magnetic NPs induced by the magnetic field, the generation of ROS, and nutrient starvation induced by GOx. The biomass and average thickness of *Escherichia faecalis* and *C. albicans* biofilms were both decreased after culturing with GMNPs for 48 h, and surface volume ratio of biofilms was increased. In another case, Elbourne *et al.* found that Ga-Fe metal droplets could be actuated by low-intensity rotating magnetic fields and formed sharp edges to combat bacteria and biofilm (Figure 4A) (Elbourne et al., 2020). Schematic diagram, SEM images and TEM images indicated that the magnetic field changed Ga-Fe droplets into spheroids, nanorods, and nanostars (Figures 4B–E). After the treatment mediated by Ga-Fe droplets with a magnetic field, the structure of biofilm was obviously damaged, and the membrane of bacteria was also penetrated and destroyed (Figures 4F, G). About 99% of live bacteria area found dead after the coincubation of bacteria with NPs and exposure to a magnetic field for 90 min. Besides, red blood cell (RBC), white blood celllysis testing, hemolysis percentage and platelet aggregation tests indicated the Ga-Fe droplets did not significantly change the concentration of WBCs and white blood cell or induce hemolysis, or lead significant platelet aggregation. Magnetophysical strategy is a novel therapy method; however, its effective sterilization takes too long, which limits its potential clinical application.

##### 3.1.3.2 Magnetothermal therapy

In addition to magnetophysical therapy, some metal materials like Fe and Co will align directionally when they are exposed to a magnetic field, and hysteresis loss/relaxation loss will generate heat energy, this process is called the magnetothermal effect, Fe and Co based nanomaterials have been applied for bacterial infection via the magnetothermal effect. Hatamie et al. synthesized graphene/Co/poly(ethylene glycol) (PEG) nanocomposites to kill bacteria (Singh et al., 2015; Hatamie et al., 2020). Under alternative current magnetic field, heat generated by Co and the wind edge of graphene (rGO) had synergistic germicidal effect, the rGO/Co/PEG nanocomposites showed antibacterial activity toward *E. coli* bacteria of about 100% within 15 min. Bigham and his co-workers constructed a magnetic Mg_2_SiO_4_-CuFe_2_O_4_ nanocomposite (Bigham et al., 2019). Thanks to the magnetothermal and bactericidal effect of Cu ions, these disks showed inhibitory effects against both *S. aureus* and *E. coli* (inhibition zones against bacteria around disks are 8.2 ± 0.25 mm and 30.8 ± 0.2 mm, respectively). Besides, this multifunctional ceramic also had the effect of promoting bone regeneration. At present, there are limited materials with magnetothermal effects. Developing more materials with magnetothermal effects may be helpful for the development of this research direction.

### 3.2 Microenvironment stimuli-responsive strategy

The formation of an infectious microenvironment is complicated, its unique features include hypoxia, low pH, enriched redox products, generous toxins, and abundant enzymes (Hu et al., 2022b). Many nanomaterials change these characteristics, and scientists have utilized the interaction between these nanomaterials and infectious microenvironments to construct many intelligent nanomedicines with high spatiotemporal controllability.

#### 3.2.1 Hypoxia-responsive strategy

**FIGURE 5:**
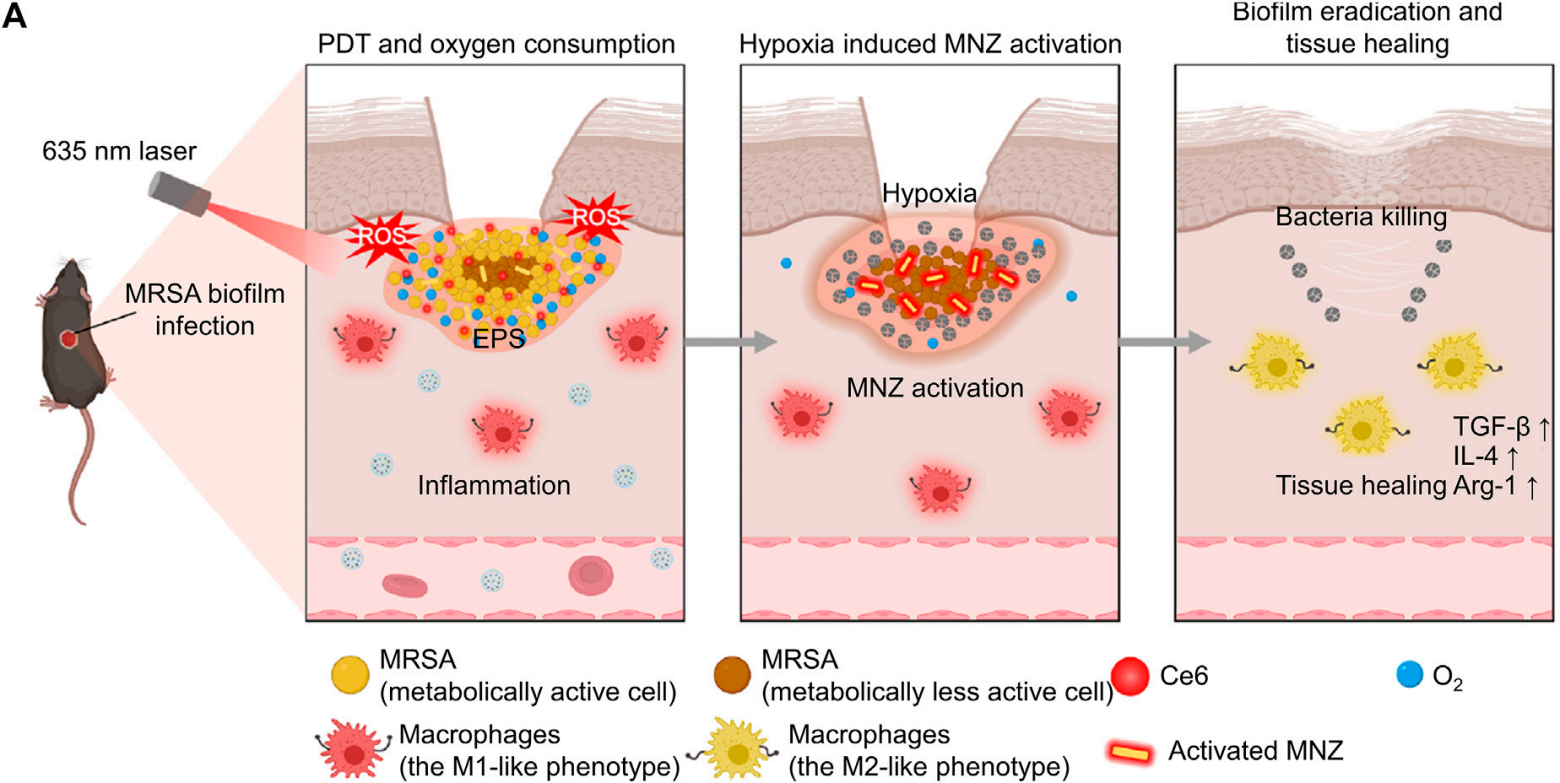
**(A)** The schematic diagram shows the process of the nanosystem exerting PDT effect, the nanosystem releasing antibiotics for long-term sterilization in hypoxic environments, and the nanosystem promoting tissue healing (Xiu et al., 2022; Xiu et al., 2022). Copyright 2022 Springer nature.

Due to the continuous oxygen consumption by aerobic bacteria and facultative anaerobic and the low permeability of the infected microenvironment, the oxygen content in the infected microenvironment is insufficient (Wang et al., 2022a). Hypoxic microenvironment induces the overexpression of many reductases, such as azoreductase and nitroreductase, so researchers can design corresponding enzymes-responsive nanomaterials to target the hypoxic microenvironment (Ma et al., 2019; Lin et al., 2022). For example, Ding et al. customized lactose-modified azocalix[4]arene (named LacAC4A) loaded with ciprofloxacin. Lactose provided the nanocomposite with targeting ability towards the bacterial surface, and azocalix could control the release of ciprofloxacin under the hypoxia microenvironment (Li et al., 2022). In another work, Wang’s group cleverly linked PDT and the expression of nitroreductase, rapid sterilization and long-term antibacterial effects (Xiu et al., 2022). Specifically, PDT mediated by Ce6 could rapidly produce ROS to achieve rapid sterilization, and PDT also exacerbated oxygen consumption, which promoted the expression of nitroreductase and the activation of metronidazole to combat bacteria for a long time (Figure 5A). After the treatment by nanocomposite, the bacterial biofilm was significantly damaged, and the corresponding number of bacteria decreased by about six logarithms. As an important feature of the infectious microenvironment, hypoxia brings many physiological changes, and scientists can utilize these changes to design more sophisticated nanomedicines.

#### 3.2.2 pH-responsive strategy

**FIGURE 6:**
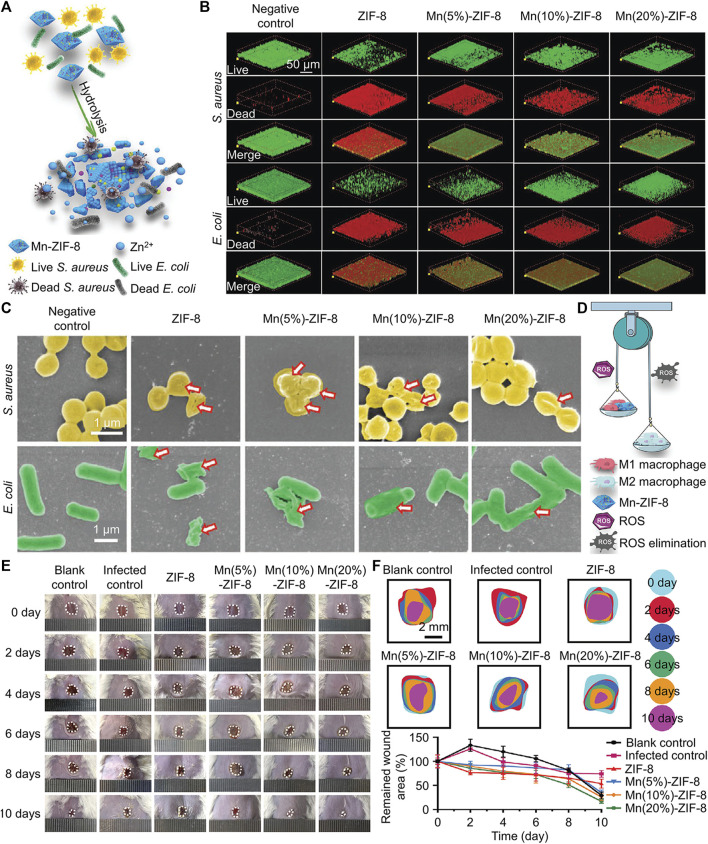
**(A)** Schematic diagram of the antibacterial mechanism of Mn-ZIF-8. **(B)** Live/Dead images of different biofilm after treated by Mn-ZIF-8. **(C)** SEM images of different biofilm after treated by Mn-ZIF-8. **(D)** Schematic diagram of the antiinflammation mechanism of Mn-ZIF-8. **(E)** Images of skin wounds under different treatments. **(F)** Changes in the sizes of wound areas in different groups (Wan et al., 2021). Copyright 2021 Wiley-VCH.

Bacteria produce acidic metabolites such as lactic acid, which lower the pH value of the infected microenvironment (Ma et al., 2020). There are two main types of pH-responsive nanomaterials: materials that can break chemical bonds in acidic environments and materials with ionizable functional groups. For instance, Wan and her co-workers created a novel Mn-doped zeolitic imidazolate framework-8 (named Mn-ZIF-8) (Wan et al., 2021). ZIF-8 was easily disintegrated under acidic conditions (Figure 6A), and subsequently released Zn^2+^, Mn^2+^ and Mn^4+^ from inside (Wan et al., 2021). As 3D Live/Dead (Figure 6B) and SEM (Figure 6C) images are shown, Zn^2+^ induced bacterial death and biofilm destruction via generating ROS. Mn^2+^ and Mn^4+^ could exert the effects of various nanoenzymes and promote the polarization of macrophages from pro-inflammatory M1 type to anti-inflammatory and repair-promoting M2 type (Figure 6D). Benefiting from the comprehensive antibacterial and anti-inflammatory effects of Mn-ZIF-8, bacterial infected wounds can heal within 10 d (Figures 6E, F). All biocompatibility tests indicated that Mn-ZIF-8 had good cytocompatibility, hemocompatibility and histocompatibility, which showed its promising potential for clinical translation. Unlike the above study, Xu et al. (2022) modified Fe_3_O_4_ layer by layer with polydopamine, Ag, polydopamine, and glycol chitosan separately (named FePAgPG). In a neutral environment, FePAgPG did not have targeting ability, but in an acidic environment, glycol chitosan could be protonated and bring positive the charge to the nanoplatform, which provided FePAgPG with targeting ability towards the bacterial membrane. The polydopamine located in the second layer prevented the premature release of Ag^+^ from causing damage to normal tissues and cells, and the great PTT effect of polydopamine could also combat biofilm effectively. Moreover, it’s practical that because this nanocomposite contained Fe_3_O_4_, it could be easily attracted and removed by a magnet after being applied to dental caries, which provided convenience for the clinical operations of dentists.

#### 3.2.3 Redox products-responsive strategy

##### 3.2.3.1 ROS-responsive strategy

Unlike the normal tissue microenvironment, there is a large amount of ROS in the bacterial infection microenvironment (Hicks et al., 2019). This is because bacteria will secrete lipopolysaccharide, DNA, peptidoglycan, flagellin and other proinflammatory substances to promote the recruitment of inflammatory cells, which will produce ROS (Suzuki et al., 1996; Graves, 2008; Woo et al., 2022). Scientists have developed various polymer nanomaterials for ROS-responsive antibacterial strategy, on the one hand, this strategy achieves precise and on-demand treatment of drugs, on the other hand, this strategy consuming ROS to prevent its damage to the host. Mesoporous silica nanoparticles (MSNs) have a high specific surface area, high porosity, and a radially ordered porous structure, makes them widely applied in drug delivery therapy, but MSNs-mediated drug delivery strategy lacks the ability for controllable delivery (Mizoshita and Tanaka, 2017). To solve this problem, Li et al. (2022) adopted thioketal grafted methoxy PEG (mPEG-TK) as the intelligent door control system of MSNs to carry vancomycin. TK could degrade polymers under ROS stimulation and release vancomycin by opening the door of MSNs. The experimental results showed that with the increase of ROS, the release of vancomycin significantly accelerated, and this process lasted for 36 h. The bacterial infection was completely eliminated after 14 d of treatment with this new antibiotic carrier.

##### 3.2.3.2 GSH-responsive strategy

As the infection microenvironment presents hypoxia, bacteria need to carry out glycolysis, which will produce a large amount of GSH (Liu et al., 2021). In addition, as mentioned earlier, there is a high level of ROS in the infected microenvironment. Bacteria will produce GSH to consume ROS to resist the damage caused by ROS (Wang et al., 2021b). However, there are relatively few materials that can respond to GSH. Until recent years, scientists attempted to construct GSH-responsive nanomaterials via unstable disulfide bonds. Li et al. (2021b) developed mesoporous organosilica NPs to deliver Ag and gentamicin. In order to prevent the uncontrolled release of antibiotics and Ag from causing toxicity to normal tissues, the authors introduced disulfide bonds to modify this nanocomposite. The controlled release mediated by disulfide bonds could achieve the efficient utilization of Ag by inhibiting its aggregation, while the on-demand release of gentamicin promoted the entry of Ag into bacteria. The synergistic effect between Ag and gentamicin was significantly greater than their respective effects on bacteria. More importantly, this new nano-drug exhibited strong killing effects on multiple drug-resistant bacteria. GSH-responsive disulfide bonds have been widely used in other antibacterial gas and drug therapies, and we will not elaborate on these here (Peng et al., 2022; Su et al., 2022). However, disulfide bond is a well-known unstable chemical bond that can be broken by ROS, nitric oxide and other stimuli, which may lead to the low sensitivity of disulfide bond-mediated controlled release system because it is easily affected by other factors in the complex infected microenvironment (Wang et al., 2021c; Yang et al., 2021).

#### 3.2.4 Toxin-responsive strategy

**FIGURE 7:**
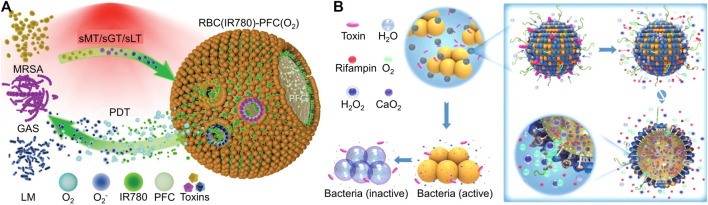
**(A)** Schematic diagram of biomimetic nanobubbles for the on-demand PDT towards bacterial infections (Zhuge et al., 2022). Copyright 2022 Wiely-VCH. **(B)** Schematic illustration of stimulus-powered antibiotic release from nanoreactors for bacterial infection therapy (Wu et al., 2019). Copyright 2019 Springer nature.

During bacterial colonization and proliferation, bacteria secrete various toxins, and the amount of toxins is often positively correlated with bacterial vitality and the infection process. For example, bacteria secrete toxins and cause damage to the host cell membrane, which inspired scientists to design many toxin-responsive nanomedicines (Pornpattananangkul et al., 2011). Chen’s group designed a bacterial toxin-responsive nanoplatform to achieve on-demand and efficient PDT against bacterial infection (Zhuge et al., 2022). Specifically, PDT often causes damage to host cells, and PDT relies heavily on oxygen content, but the bacterial infection microenvironment exhibits characteristics of hypoxia. To solve these two scientific problems, the authors employed RBC membranes to co-encapsulate perfluorocarbon nanoemulsion and PSs. The pore-forming toxin secreted by *methicillin-resistant S*. *aureus*, group A *Streptococcus*, and *Listeria* monocytogenes (LM) caused damage to the RBC membrane, which led to the release of oxygen and PSs in perfluorocarbon nanoemulsion (Figure 7A). The on-demand release of PSs combined with sufficient oxygen had achieved a good antibacterial effect in the vicinity of bacterial infections. Similarly, Wu and his co-workers utilized liposomes to coat CaO_2_ and antibiotics (Wu et al., 2019). Under the action of bacterial toxin, the outer shell of the liposome ruptured, and then water molecules entered the interior of the liposome through the perforation and reacted with CaO_2_ to produce oxygen. The large amount of oxygen facilitated the rapid release of antibiotics, which exerted an antibacterial effect (Figure 7B). Toxin-responsive nanomaterials are almost based on the interaction between toxins and lipids. In the future, researchers can design more intelligent nanomaterials based on the interactions between other toxins and materials.

#### 3.2.5 Enzyme-responsive strategy

Bacteria secrete multiple overexpressed enzymes in the bacterial infection microenvironment, such as matrix metalloproteinases (MMPs), lipase and gelatinase (Zhou et al., 2022). These enzymes cause the decomposition of certain materials, and researchers can use these materials degraded by these enzymes to construct enzyme-responsive NPs.

The wound healing is inhibited by bacterial infections and bacterial secretion of MMPs (Eming et al., 2008). To treat bacterial biofilm-induced corneal blindness, Han *et al.* MMP-responsive supramolecular nanoplatform, which was composed of Ce6, β-cyclodextrin (β-CD) and MMP-9-sensitive peptides (Han et al., 2020). In the microenvironment without MMP-9, the surface of NPs carried negative charges, which could prevent NPs from adhering to normal eye cells and thus prolong their retention time in tears. Under the stimulation of MMP-9, the negatively charged peptide shell was destroyed, and positively charged components within NPs were exposed and targeted negatively charged bacterial biofilms via electrostatic attraction. NPs with the ability to target biofilms effectively enhanced the antibacterial effect of Ce6, which prevented further damage to the cornea by bacteria and effectively alleviated corneal inflammation.

Many Gram-positive and Gram-negative bacteria secrete lipase such as *Bacillus*, *Pseudomonas*, and *Burkholderia* (Bender and Flieger, 2010). Vasilev’s group utilized lipase-responsive polycaprolactone to load ultra-small Ag NPs (1.6 ± 0.2 nm) for selective combating bacteria (Uroro et al., 2023). Polycaprolactone is a biocompatible material approved by the Food and Drug Administration (FDA) for application in the medical field. Under the action of lipase secreted by bacteria, Polycaprolactone could decompose and release antibacterial Ag^+^. In addition, this nanoplatform eliminated bacteria that secreted lipase and did not exert an inhibitory effect on bacteria that did not secrete lipase, such as *Escherichia coli*, the characteristic of this nanoplatform is conducive to the efficient removal of target pathogenic bacteria without disrupting the balance of bacterial communities. Wang et al. (2022b) constructed a nanodrug with a core loaded with alpha-lipoic acid and a poly amidoamine dendrimer shell loaded with minocycline hydrochloride. Under the action of an acidic microenvironment and lipase, NPs released minocycline hydrochloride and alpha-lipoic to combat bacteria and resist the oxidation of ROS for comprehensively treating periodontitis.

Xia’s group developed an anti-infection microneedle patch based on the characteristic of *Staphylococcus aureus* secretes gelatinase (Lei et al., 2022). The tip of this microneedle was composed of degradable materials polyethylene pyrrole and Type III collagen protein, which could achieve exogenous delivery of gelatin nanocomposite. Then, the gelatin nanocomposite would release antibacterial photothermal peptide AMP-Cypate under the stimulation of gelatinase. Experimental data showed that the microneedle patch could degrade and release gelatin nanocomposite within 20 min, and the efficiency of gelatin nanocomposite releasing antibacterial photothermal peptide increased with the rise of bacterial concentration. This microneedle patch cleverly combined non-invasive microneedle therapy, PTT and on-demand drug delivery strategy. In another study, Liu’s group used RBC membrane and gelatin to deliver Ru-Se NPs (Lin et al., 2019). RBC membrane prevented NPs from being eliminated by the body and neutralize exotoxin produced by bacteria. The release of Ru-Se NPs from gelatin could effectively damage the bacterial cell membrane, leading to leakage of its contents. More importantly, Ru-Se NPs had fluorescence imaging functions, which could help scientists monitor the treatment process in real time.

Although researchers have developed many enzyme-responsive antibacterial nanomaterials in recent years, an issue that cannot be ignored is that other components generated in nanomaterials may inactivate enzymes (such as ROS and heat). Therefore, how to construct enzyme-responsive antibacterial NPs without affecting enzyme activity is a future scientific problem.

Advances in precise antibacterial therapeutics based on stimuli-responsive nanomaterials are listed in Table 1.

**TABLE 1:** Advances in precise antibacterial therapeutics based on stimuli-responsive nanomaterials.

Type of stimuli		Nanocomposite	Responsive groups	Model bacteria	Antibacterial mechanisms	Advantages	Disadvantages	Ref.
Exogenously stimulated	Light and US	mTiO_2_@PDA	PDA and TiO_2_	*S. aureus* and *E. coli*	PDT, PTT and SDT	Rich in functions and stable	Need two kinds of stimuli	Cheng et al. (2023b)
	Light	F@Ce6-M	Ce6 and Coumarin 6	*P. gingivalis*, *F. nucleatum* and *S. gordonii*	PDT	High penetration and generate oxygen	Complex structure	Sun et al. (2021)
	Light	GNRs@mSiO_2_-SNO/ICG	Au nanorod and ICG	*P. gingivalis*, *F. nucleatum* and *S. gordonii*	PDT, PTT and NO	Rich in functions and regulate inflammation	Gas diffusion and high cost	Qi et al. (2022)
	US	DT-Ag-CS^+^	TiO_2_	*P. gingivalis*	SDT and chitosan	Robust ROS generation	Low sterilization rate	Xin et al. (2023)
	Magnetic	GLM-Fe	Fe	*S. aureus* and *P. aeruginosa*	Sharp edge	Effectively remove biofilm	Requires long-term action	Elbourne et al. (2020)
	Magnetic	Fe_3_O_4_-ZnO	Fe_3_O_4_	*S. aureus* and *E. coli*	Magnetothermal and ZnO	Clean water and high biosafety	Need AC magnetic field	Singh et al. (2015)
	Magnetic and light	rGO/Co/PEG	Co and rGO	*E. coli*	Magnetothermal and PTT	Dual hyperthermia therapy	Need more data of sterilization	Hatamie et al. (2020)
	Magnetic	Mg_2_SiO_4_-CuFe_2_O_4_	CuFe_2_O_4_	*S. aureus* and *E. coli*	Magnetothermal and Cu ion	Rich in functions and long-term sterilization	Low biosafety	Bigham et al. (2019)
Microenvironmentally responsive	Hypoxia	Cip@LacAC4A	Azocalix	Multidrug-resistant *Pseudomonas aeruginosa*	Ciprofloxacin	Long-term sterilization	Low anti-biofilm rate	Li et al. (2022)
	Hypoxia and light	HA-Ce6-MNZ	Metronidazole and Ce6	Methicillin-resistant *S. aureus* (MRSA)	Metronidazole and PDT	Long-term sterilization and high sterilization rate	Low PDT effect	Li et al. (2022)
	pH	Mn-ZIF-8	ZIF-8	*S. aureus* and *E. coli*	Zn ion	Rich in functions and simple structure	Not stable	Wan et al. (2021)
	pH	FePAgPG	Glycol chitosan	*S. mutants*	PTT and Ag ion	Easy to remove	Complex structure	Xu et al. (2022)
	ROS	Van-mPEG-TK-MSNs	Thioketal	*S. aureus*	Vancomycin	Long-term sterilization	Low sterilization rate	Li et al. (2020)
	GSH	Ag-MONs@GEN	Disulfide	*E. coli*, *P. aeruginosa*, *S. aureus* and *E. faecalis*	Gentamicin and Ag ion	High biosafety	High cost	Li et al. (2021b)
	Toxin	RBC(IR780)-PFC	RBC membrane	MRSA, group A *Streptococcus*, and *Listeria* monocytogenes	PDT	Rich in functions and on-demand therapy	Gas diffusion	Zhuge et al. (2022)
	Toxin	RFP-CaO_2_@PCM@Lec	Mixture of two fatty acids and a liposome coating	MRSA and *B. subtilis*	Antibiotic	Generate oxygen	Produce toxic H_2_O_2_	Wu et al. (2019)
	Enzyme	Ad-MMP-S PEPs	MMP-9-sensitive peptides	*P. aeruginosa* and *S. aureus*	PDT	High sterilization rate	Not stable	Han et al. (2020)
	Enzyme	pAgNCs@PCL	Polycaprolactone	*S. aureus*, *E. coli* and *P. aeruginosa*	Ag nanoclusters	Selective sterilization	Not stable and high cost	Uroro et al. (2023)
	Enzyme	Ru-Se@GNP-RBCM	Gelatin	MRSA and *E. coli*	Se NPs	Rich in functions	Complex structure	Lin et al. (2019)

## 4 Challenges and future perspectives

In the post-antibiotic era, nanomaterials are regarded as potential substitutes for antibiotics due to their high bactericidal efficiency, rich functionality, and ease of modification. Especially stimuli-responsive nanomaterials based on the physical and chemical properties of the nanomaterials themselves and the characteristics of the infectious microenvironment, the antibacterial effects or release of antibacterial agents of these nanomaterials can be effectively controlled to achieve precise antibacterial treatment. Intelligent stimuli-responsive nanomaterials reduce toxicity to tissues and improve antibacterial efficiency, and they have been widely developed and achieved satisfactory results in recent years. However, there are still many challenges to be addressed in applying stimuli-responsive nanomaterials in antibacterial therapy. (1) Nanomaterials utilize various physical effects, chemical effects and antibacterial agents to kill bacteria, but these ways inevitably cause damage to normal tissue cells, which exacerbates inflammatory reactions and inhibits the healing of infected tissues (Bai et al., 2023). More advanced stimuli-responsive nanomaterials should have the function of regulating inflammatory reactions or promoting tissue healing. For example, scientists can focus on cells that regulate inflammation and promote tissue repair, such as macrophages and regulatory T cells. (2) Although the existing stimuli-responsive nanomaterials have rich functions, their synthesis steps must be simplified, making it difficult for others to replicate experimental results and conduct clinical transformation (Lu et al., 2016). Therefore, scientists need to design stimuli-responsive nanomaterials with simple synthesis steps that do not require strict experimental conditions. (3) The existing stimuli-responsive nanomaterials have high costs and strict storage conditions (Gu et al., 2016). Therefore, cheaper, stable, and easily obtainable materials should be more widely used in constructing stimuli-responsive nanomaterials. (4) Life activities have the characteristics of complexity and variability, the metabolic patterns of nanomaterials in the body are still unclear, and the diseases' fate also needs to be explored (Couvreur and Vauthier, 2006). Therefore, even though nanomaterials have been approved for clinical treatment, they still need some help. We should obtain more data from animal experiments and preclinical studies about stimuli-responsive nanomaterials. (5) Reduction in size of nanomaterials makes them deposit in the main organs, which affects the biocompatibility of nanomaterials and causes cytotoxicity. In addition, more complex nanomedicines in recent years will also impact on their safety. However, current preclinical toxicity experimental data cannot represent the real situation of the human body. Microfluidics technologies provide cytotoxicity tests with more realistic real microenvironments, they can improve the reliability of cytotoxicity tests (Beckwith et al., 2018). (6) The further commercial prospects of these strategies are of great significance for their sustainable development (Rao et al., 2018). However, there are many challenges currently hampering their commercial prospects, including scalable manufacturing, biocompatibility, regulation and socioeconomic acceptance. The solution to this problem demands collaboration among materials scientists, pharmacists, clinicians, funding bodies, governments and big companies.

## 5 Conclusion

In conclusion, stimuli-responsive nanomaterials and their antibacterial strategies have been widely proven in sterilization fields. Next, further application of stimuli-responsive nanomaterials to large animal models is necessary. Afterwards, human trials and FDA approval are needed for further application of stimuli-responsive nanomaterials. Finally, stimuli-responsive nanomaterials enter the stage of commercial exploration and mass production. Although stimuli-responsive nanomaterials face many challenges in their clinical application, they are still great substitutes for antibiotics. With the advancement of technology, in-depth research exploration, and the improvement of relevant laws and regulations, the application of stimuli-responsive nanomaterials in bacterial infection will inevitably have a bright prospect.
